# The clinical features of severe COVID-19 with respiratory failure: A Chinese single-center retrospective study

**DOI:** 10.1097/MD.0000000000036110

**Published:** 2023-12-01

**Authors:** Guosheng Liu, Chunhong Du, Weicheng Du, Deyuan You

**Affiliations:** a Department of Critical Care Medicine, The Second Affiliated Hospital of Fujian Medical University’s, Quanzhou, China.

**Keywords:** COVID-19, epidemiology, outcome, respiratory failure

## Abstract

The global pandemic of COVID-19, caused by the novel coronavirus SARS-CoV-2, has resulted in widespread alterations to public health measures worldwide. This observational study aimed to assess the clinical features and results of respiratory failure in patients with severe COVID-19. A single-center observational study was performed at a Chinese hospital between November 1, 2022, and February 31, 2023. All 182 enrolled patients were diagnosed with respiratory failure, 84 patients were infected with COVID-19, and the other 98 patients were not infected. A review of available medical records at admission and discharge, including neuroimaging, laboratory values at admission, mortality, length of hospitalization, and hospital costs, was conducted during the COVID-19 pandemic. All 182 eligible patients completed the follow-up. There was no significant difference in baseline characteristics between respiratory failure combined with COVID-19 (*P* > .05). Respiratory failure combined with COVID-19 infection may lead to higher 30-day mortality (16.36% vs 7.14%, *P* = .005), longer hospital stays (22.5 ± 5.9 vs 12.8 ± 4.2, *P* < .001), larger hospitalization costs (*P* < .001), and increased hospitalization complications, such as pulmonary embolism (10.30% vs 4.76%, *P* = .039), deep vein thrombosis (33.33% vs 18.57%, *P* = .001), incidence of 7-day delirium (69.70% vs 46.19%, *P* < .001), and respiratory failure (38.18% vs 24.77%, *P* = .005). If respiratory failure occurs while the patient is infected with COVID-19, treatment and prognosis worsen. Our understanding of COVID-19 and the care we provide to patients with respiratory failure is crucial to better prepare for a potential pandemic.

## 1. Introduction

The COVID-19 pandemic, caused by the new coronavirus SARS-CoV-2, has led to significant alterations in public health strategies, resulting in numerous fatalities worldwide.^[1]^ For nearly 3 years, the healthcare system has been confronted by the COVID-19 crisis, impacting the global management, and treatment of patients in intensive care units. China adopted a unique strategy in handling the outbreak before December 2022, starting with the Wuhan lockdown in 2020 and implementing a dynamic zero-COVID policy. They utilized precise measures for prevention and control to halt the spread of SARS-CoV-2, distinguishing themselves from other nations.^[2]^ The world’s attention has been captured by China’s rapid epidemic outbreak, and it is anticipated that China will modify its prevention and control measures by December 2022. China’s healthcare sector has been greatly impacted by the sudden emergence of SARS-CoV-2, causing significant disruption and even the complete breakdown of medical infrastructure. Hospitals have admitted a significant influx of COVID-19 patients. Nonetheless, limited data exist regarding the potential for severe illness linked to numerous comorbidities. When combined with COVID-19 infection, respiratory failure can become a severe respiratory illness, leading to a substantial rise in mortality rates.^[3,4]^

Respiratory failure is mainly due to altered ventilation and/or perfusion and the inability to maintain proper gas exchange to meet normal cellular metabolism. The pathological features of SARS-CoV-2 pulmonary injury include widespread damage to the alveoli and the formation of blood clots. In addition, nosocomial bacterial superinfections and ventilator-induced lung injury may also occur.^[5]^ Patients experiencing external respiratory failure will endure increased lung damage due to contracting COVID-19. Hraiech^[6]^ reported that during the widespread COVID-19 epidemic in France, the risk of undocumented persons entering the NICU and developing acute respiratory distress syndrome (ARDS) increased significantly. A previous study also reported that infection with SARS-CoV-2 causes COVID-19, and respiratory failure is the principal clinical presentation and the main cause of death.^[7]^ The epidemic of COVID-19 has caused acute lung injury to millions of people worldwide; approximately 5% of infected patients are severe patients who are prone to cytokine storms, ARDs, respiratory failure, shock, organ system failure, etc, and the mortality rate exceeds 30%.^[8]^ Michalski^[9]^ also reported that although COVID-19 is a multisystem disease, SARS-CoV-2 mainly infuses and damages lung organs causes pneumonia, and, in severe cases, can lead to ARDS and respiratory failure.

Nevertheless, there have been no investigations conducted on the epidemiology and medical characteristics of respiratory failure in China amidst the SARS-CoV-2 outbreak.

Hence, our study aimed to examine how the SARS-CoV-2 pandemic has affected the shared risk factors and treatment strategies for individuals experiencing respiratory failure.

## 2. Materials and methods

### 2.1. Study design and patients

A single-center observational study was conducted at our hospitals in China from November 1, 2022, to February 31, 2023. To be included, patients had to meet the following criteria: (1) were diagnosed with respiratory failure upon admission by 2 intensivists and met the diagnostic criteria of PaO_2_ < 60 mm Hg and PCO_2_ > 50 mm Hg. (2) Patients aged 18 to 90 years. The exclusion criteria included (1) a patient whose recovery was not expected upon admission; (2) pregnant women and patients; (3) multiple organ dysfunction; and (4) other explanations identified by researchers. The patients were categorized into 2 groups based on their COVID-19 infection status. Out of the 219 individuals with respiratory failure, 182 patients who met the criteria were enrolled. Data from patients of all ages with respiratory failure who presented to our center were collected. We examined illustrated information obtained from current medical records regarding factors that increase the likelihood of harm (gender, age, cause of injury, etc). and administration. The follow-up will be carried out via online or telephone communication. The study procedures were formulated and carried out to assess the possible safety and effectiveness in individuals with respiratory failure following the outbreak of the SARS-CoV-2 virus. The registration number FJEM-IPR-2022901 (date 16/Nov 2022) was obtained with protocol approval from the Clinical Research Ethics Committees of the Second Affiliated Hospital of Fujian Medical University (Approval number FJMU-2022-108).

### 2.2. Statistical analysis

The unpaired *t*-test was used to analyze normally distributed continuous data (mean ± SD), while the independent-samples Mann–Whitney *U* test was used to analyze nonnormally distributed data. Furthermore, the χ2 test or the χ2 test with continuity correction was employed for comparing the categorical data. Statistically significant mean differences or risk ratios were calculated, taking into account two-sided 95% confidence intervals and *P* values < 0.05. IBM SPSS Statistics version 24 for Windows (IBM, Chicago, IL) was used to conduct the statistical analysis. The assessments did not include any interim analysis. The Clinical Research Ethics Committee from the Second Affiliated Hospital of Fujian Medical University conducted data supervision.

## 3. Results

### 3.1. Baseline patient characteristics: overall population

**Figure 1.**
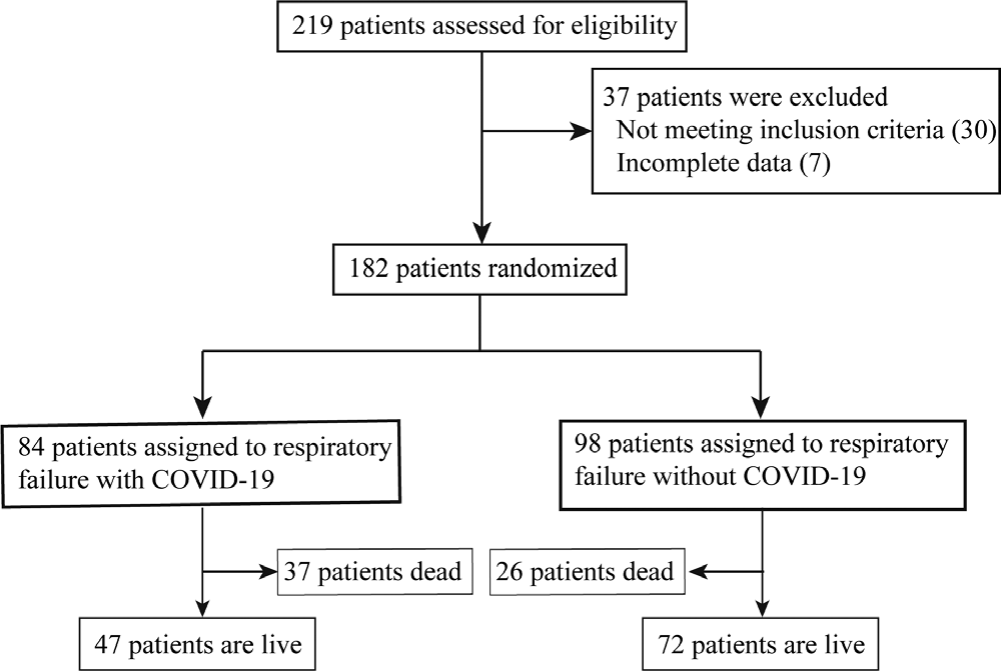
Flow chart.

Between November 1, 2022, and February 31, 2023, a comprehensive evaluation was conducted on a total of 219 individuals. Real time quantification-polymerase chain reaction was utilized to conduct COVID-19 testing on patients. COVID-19 was present in 84 patients who experienced respiratory failure, while the remaining 98 patients had respiratory failure but not due to COVID-19 (Fig. 1). In the group with COVID-19 infection, the average age was 67.3 ± 9.7 years (ranging from 18 to 90 years), while in the group without COVID-19, the average age was 66.8 ± 9.4 years (ranging from 19 to 89 years). Additionally, there was no notable distinction observed in the baseline characteristics (Table 1) when comparing respiratory failure in conjunction with COVID-19 infection to cases without it.

**Table 1:** Comparison of baseline data.

	COVID-19 (n = 84)	Non- COVID-19 (n = 98)	*P*
Age (Y, mean ± SD)	67.3 ± 9.7	66.8 ± 9.4	.7225
*Gender, no. (%*)			.981
Male	49 (58.33%)	57 (58.16%)	
Female	35 (41.67%)	41 (41.84%)	
BMI (kg/cm^2^, mean ± SD)	23.58 ± 4.97	24.09 ± 5.39	.510
Heart rate (bpm)	89.67 ± 12.23	88.15 ± 12.19	.403
Respiratory rate (bpm)	21.82 ± 4.54	22.19 ± 4.40	.578
PaO_2_ (mm Hg)	56.78 ± 9.21	55.94 ± 9.08	.537
PaCO_2_ (mm Hg)	81.05 ± 15.37	80.28 ± 14.89	.732
Blood pH value	7.32 ± 1.71	7.29 ± 1.68	.905
MBP (mm Hg)	98.93 ± 18.22	99.61 ± 19.27	.808
OI (mm Hg)	143.65 ± 32.38	146.70 ± 31.84	.524
*Smoking history, no. (%*)			.653
Yes	35 (41.67%)	45 (45.92%)	
No	49 (58.33%)	53 (54.08%)	
*Drinking history, no. (%*)			.766
Yes	37 (44.05%)	41 (41.84%)	
No	47 (55.95%)	57 (58.16%)	
*Living environment, no. (%*)			.756
Town	53 (63.10%)	65 (66.32%)	
Countryside	31 (36.90%)	33 (33.68%)	
*Past medical history, no. (%*)			
Hypertension	49 (58.33%)	52 (53.06%)	.550
Hyperlipidemia	41 (48.81%)	37 (37.76%)	.137
Diabetes	30 (35.71%)	33 (33.67%)	.876
Heart disease	12 (14.29%)	15 (15.31%)	1.000
Chronic kidney disease	15 (17.86%)	14 (14.29%)	1.000
Cerebrovascular disease	23 (27.38%)	27 (27.55%)	1.000

### 3.2. The clinical outcome between the 2 groups

**Figure 2.**
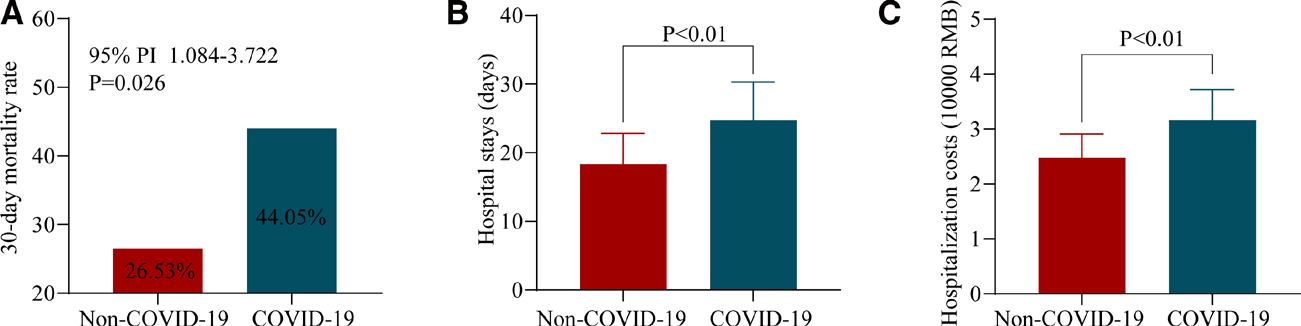
The difference in clinical outcomes between the 2 groups.

Respiratory failure patients might experience higher mortality rates due to COVID-19 infection. We analyzed the disparities between the 2 groups. In the group of patients with respiratory failure combined with COVID-19 infection, the mortality rate within 30 days was 44.05% (37 out of 84), whereas, in the group of patients with respiratory failure without COVID-19 infection, the mortality rate within 30 days was 26.53% (26 out of 98), as shown in Figure 2A. The COVID-19-infected group showed a significantly higher 30-day mortality rate than the group without COVID-19 infection (*P* = .026, Fig. 2A). Higher total 30-day mortality was linked to the combination of COVID-19 and respiratory failure in terms of clinical outcomes.

### 3.3. Hospital stays and hospitalization costs

Among patients with respiratory failure and COVID-19 infection, the mean length of hospitalization was 24.7 ± 5.6 days, whereas non-COVID-19-infected patients had an average stay of 18.3 ± 4.5 days, showing a statistically significant disparity (*P* < .001, Fig. 2B). The average hospitalization cost for patients infected with COVID-19 was 31,600 RMB, which was significantly higher than the cost for non-COVID-19 patients (24,800 RMB) (*P* < .001, Fig. 2C). As a result, the hospital stays and costs for patients with respiratory failure have been greatly elevated due to COVID-19 infection.

### 3.4. Complications between the 2 groups

While undergoing treatment for respiratory failure, we additionally observed a higher incidence of complications among patients afflicted with COVID-19. The occurrence of pneumonia was higher in patients infected with COVID-19 than in those not infected with COVID-19, although there was no statistically significant difference between the 2 groups (89.29% vs 81.63%, *P* = .148, Table 2). In COVID-19-infected individuals, we observed an increase in the occurrence of pulmonary embolism (9.52% compared to 2.04%, *P* = .046) and deep vein thrombosis (34.52% compared to 18.36%, *P* = .013). Moreover, there was a significant statistical distinction between the 2 groups (*P* < .05, Table 2). Furthermore, it was also observed that the occurrences of delirium lasting for 7 days (60.71% vs 43.88%, *P* = .023) and neurological impairments (35.71% vs 21.43%, *P* = .032) were higher in patients infected with COVID-19 than in those without COVID-19. The 2 groups did not show any notable disparities in heart failure (35.71% vs 33.67%, *P* = .773), multiple organ dysfunction syndrome (26.19% vs 17.34%, *P* = .147), abnormal liver enzymes (46.43% vs 37.76%, *P* = .237), diarrhea (29.76% vs 22.45%, *P* = .261), or vomiting (19.05% vs 15.31%, *P* = .503). Table 2 displays the outcomes.

**Table 2:** Comparison of complications between 2 groups.

	COVID-19 (n = 84)	Non-COVID-19 (n = 98)	*P*
Pneumonia, no. (%)	75 (89.29%)	80 (81.63%)	.148
Pulmonary embolism, no. (%)	8 (9.52%)	2 (2.04%)	.046
DVT, no. (%)	29 (34.52%)	18 (18.36%)	.013
7 days delirium, no. (%)	51 (60.71%)	43 (43.88%)	.023
Heart failure, no. (%)	30 (35.71%)	33 (33.67%)	.773
MODS, no. (%)	22 (26.19%)	17 (17.34%)	.147
Neurological deficit, no. (%)	30 (35.71 %)	21 (21.43 %)	.032
Abnormal liver enzymes, no. (%)	39 (46.43%)	37 (37.76%)	.237
Diarrhea, no. (%)	25 (29.76%)	22 (22.45%)	.261
Vomiting, no. (%)	16 (19.05%)	15 (15.31%)	.503

## 4. Discussion

Examining the effects of the SARS-CoV-2 pandemic on common risk factors and treatment management for patients with respiratory failure, this study is conducted at a single center and is up to date. Based on the current results, we observed no notable distinction in the initial patient attributes between the 2 groups. The combination of COVID-19 and respiratory failure was linked to an increased overall mortality rate within 30 days. Hospitalization costs and duration of hospital stays were further exacerbated by COVID-19-related respiratory failure. Furthermore, the presence of COVID-19 along with respiratory insufficiency amplifies the likelihood of experiencing various complications while being hospitalized, including but not limited to pneumonia, pulmonary embolism, delirium lasting for a week, heart failure, and multiple organ dysfunction syndrome.

Healthcare systems and practitioners have been affected by the COVID-19 pandemic on a global scale. Following December, the transmission of COVID-19 escalated in China after the termination of the zero-COVID strategy. The majority of patients who were admitted to the hospital were infected with COVID-19 either before or after admission. In general, no disparities were observed in baseline characteristics, such as age, sex, and medical history, between individuals with respiratory failure and COVID-19 infection and those without. Our investigation revealed that the combination of COVID-19 and respiratory failure can lead to a more unfavorable clinical result, heightened complications, substantially prolonged hospital stays, and increased costs for patients with respiratory failure. According to Tilliridou,^[10]^ COVID-19 patients with pulmonary embolism had a greater 30-day mortality rate than those without pulmonary embolism. The risk for pulmonary complications can be heightened by the combination of multiple traumas, COVID-19 infection, and positive computerized tomography results.^[11]^ According to Driessen,^[12]^ the mortality rate during the SARS-CoV-2 pandemic was higher than that in the previous period. In the current investigation, it was also observed that a greater number of individuals who succumbed to respiratory failure had contracted COVID-19, with the primary reason for mortality being the occurrence of multiple organ failure as a result of a severe lung infection. Additionally, Htay^[13]^ revealed a significant increase in the fatality rate among peritoneal dialysis patients who contracted COVID-19 during the Delta variant outbreak.

This study also had some limitations regarding the study design. First, the size of the sample was limited, and further extensive studies are required to investigate the influence of the SARS-CoV-2 outbreak on the typical risk elements and management of treatment for individuals experiencing respiratory distress. Furthermore, due to the nature of our cross-sectional and retrospective study, it is not possible to establish a causal relationship based on the data obtained. Therefore, future investigations focusing on the effects of a pandemic on intentional respiratory failure should consider employing a prospective, longitudinal design. This will enable the identification of risk factors and the exploration of causal connections between variables. Furthermore, this study solely focuses on the immediate negative outcomes and effectiveness of COVID-19 infection in individuals experiencing respiratory failure, emphasizing the necessity for long-term findings. One more constraint is associated with the absence of information in the medical record repository, which lacks comprehensive clinical details regarding respiratory failure, such as the extent of damage and surgical procedures performed. Hence, it is imperative to examine additional and larger groups of individuals suffering from respiratory failure due to COVID-19.

## 5. Conclusion

Based on the findings presented, patients infected with COVID-19 and experiencing respiratory failure exhibited elevated mortality rates within 30 days, prolonged hospital stays, increased hospitalization expenses, and a higher likelihood of encountering complications during their hospitalization, including pulmonary embolism and delirium lasting for 7 days. Furthermore, the implications of extended monitoring remain uncertain. Hence, it is imperative to examine additional and larger groups of individuals suffering from respiratory failure due to COVID-19.

## Author contributions

**Conceptualization:** Deyuan You, Guosheng Liu, Chunhong Du.

**Data curation:** Guosheng Liu, Weicheng Du.

**Formal analysis:** Deyuan You, Chunhong Du, Weicheng Du.

**Investigation:** Deyuan You, Guosheng Liu, Weicheng Du.

**Methodology:** Guosheng Liu, Chunhong Du, Weicheng Du.

**Resources:** Deyuan You, Guosheng Liu.

**Software:** Guosheng Liu, Weicheng Du.

**Supervision:** Deyuan You, Guosheng Liu, Weicheng Du.

**Validation:** Deyuan You, Guosheng Liu, Chunhong Du.

**Visualization:** Deyuan You, Guosheng Liu, Chunhong Du, Weicheng Du.

**Writing – original draft:** Deyuan You, Guosheng Liu, Chunhong Du, Weicheng Du.

**Writing – review & editing:** Deyuan You, Guosheng Liu.
